# Salicylic Acid and Calcium Chloride Seed Priming: A Prominent Frontier in Inducing Mineral Nutrition Balance and Antioxidant System Capacity to Enhance the Tolerance of Barley Plants to Salinity

**DOI:** 10.3390/plants13091268

**Published:** 2024-05-02

**Authors:** Rim Ben Youssef, Nahida Jelali, Cristina Martínez-Andújar, Chedly Abdelly, José Antonio Hernández

**Affiliations:** 1 Laboratory of Extremophile Plants, Centre of Biotechnology of Borj-Cédria (CBBC), P.O. Box 901, Hammam-Lif 2050, Tunisia; nahidajelali@gmail.com (N.J.); abdelly.chedly@gmail.com (C.A.); 2 Faculty of Sciences of Tunis, University of Tunis El Manar, Tunis 1060, Tunisia; 3 Group of Fruit Trees Biotechnology, Centro de Edafología y Biología Aplicada del Segura (CEBAS), Consejo Superior de Investigaciones Científicas (CSIC), 30100 Murcia, Spain; 4 Department of Plant Nutrition, Centro de Edafología y Biología Aplicada del Segura (CEBAS), Consejo Superior de Investigaciones Científicas (CSIC), 30100 Murcia, Spain; cmandujar@cebas.csic.es

**Keywords:** antioxidant enzymes, oxidative stress, seed priming, abiotic stress, crop improvement

## Abstract

The current investigation aims to underline the impact of salicylic acid or calcium chloride seed pre-treatments on mineral status and oxidative stress markers, namely levels of electrolyte leakage (EL) and lipid peroxidation levels, measured as thiobarbituric reactive substances (TBARS), and the activity of some antioxidant enzymes in roots and leaves of plants in two barley species grown under various salt treatments. Overall, our results revealed that salinity inhibits essential nutrient absorption such as iron, calcium, magnesium and potassium and stimulates the absorption of sodium. Also, this environmental constraint induced oxidative stress in plants in comparison with the control conditions. This state of oxidative stress is reflected by an increase in TBARS content as well as the stimulation of EL values. In addition, salinity induced disturbances in the activity of antioxidant enzymes, which were mainly dependent on the applied salt concentration and the species. In addition, *Hordeum marinum* maintained high antioxidant enzyme activity and low levels of oxidative stress parameters, which reinforces its salt-tolerant character. Importantly, salicylic acid or calcium chloride seed priming alleviated the mineral imbalance and the oxidative damage induced by salinity. Moreover, seed priming improves iron, calcium magnesium and potassium content and limitsthe accumulation of sodium. Also, both treatments not only decrease TBARS levels and limit EL, but they also stimulate the antioxidant enzyme activities in the leaves and roots of the stressed plants as compared with stressed plants grown from non-primed seeds. Interestingly, the beneficial effects of the mentioned treatments were more notable on *Hordeum vulgare* species.

## 1. Introduction

Salinity is one of the most urgent and critical problems in agriculture [1,2]. It already affects 20% of cultivated land and 33% of irrigated land, suggesting that salinity could affect 50% of cultivated land by 2050 [3].

As a result, this abiotic constraint presents unique difficulties for world agriculture, and it is anticipated that its detrimental effects on crop productivity will worsen [4,5]. Unbalanced mineral nutrition has been proposed as one of the harmful physiological impacts in plants exposed to high salinity [6]. It has been determined that the primary issue with nutrients during salinity may be caused by a mineral imbalance brought on by Na^+^’s competition with K^+^ and Ca^2+^ [7]. This primarily results in a nutritional imbalance that inhibits the root’s ability to absorb vital nutrients [8], and isconsidered to be one of the indicators of common metabolic disorders in salt-sensitive plants [6]. More specifically, the accumulation of Na^+^ within plant tissues, due to increased NaCl levels in the substrate, can affect their K^+^ status, thus leading to disruption of cellular function. In addition to the osmotic and ion toxicity effects, salt stress also induced oxidative stress at a subcellular level, manifested by the over-generation of reactive oxygen species (ROS), leading to a disturbance in the cell redox balance and cell damage [9]. To defend against ROS toxicity, plants possess an efficient antioxidant defense system, including enzymatic and non-enzymatic mechanisms that protect them from damaging oxidative reactions [10].

Currently, it has been shown that seed priming can significantly improve crop performance and yield in a variety of environmental circumstances. Numerous substances have the ability to function as signal transducers when under stress. For example, it has been demonstrated that, in salinity, seed priming with KNO_3_ and KCl enhanced barley germination and seedling growth. Indeed, primed seeds exhibited a higher capacity to withstand stress in comparison to unprimed ones. It was evident that these various priming treatments increased germination parameters and the essential nutrient concentration, in addition to increasing the seedling growth rate. Also, they reduced the accumulation of sodium ions and mitigated the oxidative stress of salt-induced seeds by reducing MDA levels and electrolyte leakage values [11]. Salicylic acid (SA) and calcium chloride (CaCl_2_) have been demonstrated to function as endogenous molecules that induce stress tolerance. Specifically, it has been demonstrated that pre-treating seeds with SA or CaCl_2_ strengthens their antioxidant system, thereby reducing the negative effects of salinity [12]. Thus, these effects show that these two agents could be ideal components to increase plant resistance against salinity.

One phenolic compound that can help plants cope with environmental stressors is salicylic acid (SA) [13]. Several studies have demonstrated that seed priming with SA reveals physiological process adaptations in plants exposed to various environmental stresses [14,15]. In this regard, SA functions as a natural endogenous signal molecule bycontrolling physiological and biochemical processes in defense mechanisms [16]. Numerous results indicate that SA alleviates the effects of salt stress [17,18], heat stress [19], drought stress [20] and metal stress [21]. It may have antagonistic or cooperative interactions with nutrients, growth and development of plants in both ideal and challenging conditions by reducing the buildup of Na^+^ and Cl^−^ in *Zea mays* and *Brassica* treated with salt [14]. Moreover, several works have reported that SA improved the tolerance of plants exposed to different abiotic stresses, especially salinity, by modulating antioxidant metabolism [22]. In that regard, the activities of H_2_O_2_-metabolizing enzymes (catalase and peroxidase) and superoxide dismutase (SOD) enzymes, involved in the elimination of superoxide radicals, were also modulated by SA in plants subjected to cold stress [23]. Also, it has been proposed that SA is essential to the control of SA increased *Linum usitatissimum*’s resistance to cadmium by lowering reactive oxygen species [15] and reducing lipid peroxidation to lessen the impact of salt stress and permeability of membranes [24]. It has been proposed that salt stress induced the expression of the benzoic-acid-2-hydroxylase gene, the SA biosynthetic pathway’s key enzyme in this process [25].

Seed priming with calcium seeds is another strategy to lessen the effects of salinity on plants and minimize Na^+^ and Cl^−^ injuries in plants. Indeed, Ca^2+^ has attracted a lot of attention because it appears to play a key role in many defense mechanisms that are induced by environmental stresses [26,27] and it can provide plants with a protective effect in stressed environmental circumstances. Since calcium increases antioxidant enzyme activity and decreases lipid peroxidation of cell membranes under abiotic stress, it is essential for salt stress tolerance [22]. Additionally, it has been demonstrated to preserve regular photosynthesis, stabilize cell membrane surfaces, stop solute leakage from the cytoplasm and control the metabolism of plant hormones. Furthermore, Joshi et al. (2013) showed that the beneficial response to salinity, reported in plants grown from cucumber (*Cucumis sativus* L.) seeds pre-treated with CaCl_2_, was the activation of antioxidant enzymes [28]. Additionally, previous research revealed that calcium reduced H_2_O_2_ levels, possibly through stimulating ROS-scavenging enzymes to counteract the detrimental effects of oxidative stress [29]. Several findings indicate that Na^+^ competes with Ca^2+^ for sites of binding in salinity and that Ca^2+^ directly reduces mineral toxicities. The external Ca^2+^’s mitigating effect on plants exposed to salinity may be related tothe preservation of homeostasis and an ideal K^+^/Na^+^ ratio in connection with the suppression of Na^+^ influx and K^+^ efflux, or the encouragement of K^+^ influx and Na^+^ efflux through the plasma membrane [30].

The development of crops resistant to salt is based on the identification of new salt-tolerance mechanisms. One of the most promising ways to identify new mechanisms of salt tolerance is to study physiological mechanisms in plants [4]. For this reason, the aim of the current study was to evaluate the effects of exogenous SA and CaCl_2_ on mineral nutrition and antioxidant capacity in two barley species differing in salt tolerance: *H. vulgare* L. Manel, which less tolerant to salt stress than *H. marinum* Huds. The application of the knowledge of alterations mediated by SA and CaCl_2_ may result in the discovery of new methods to enhance growth and productivity.

## 2. Results

### 2.1. Effect of SA and CaCl_2_ Seed Priming on Mineral Nutrition Status under Salinity

#### 2.1.1. Iron

The findings showed that as salt treatments increased, salinity reduced the Fe content in both species of barley (Table 1). When plants were exposed to 200 mM NaCl, the decrease became more noticeable. The NaCl-induced Fe decline reached 56% and 20.7% in roots and 58.6% and 20.3% in shoots from *H. vulgare* and *H. marinum*, respectively, compared to the control plants. Interestingly, pre-soaking seeds of both species with SA or CaCl_2_ alleviated the iron decrease in plants grown under all salt treatments as compared to unprimed plants, and the effect of SA is the one that caused a smaller decrease in Fe in the presence of NaCl (Table 1).

#### 2.1.2. Calcium

As shown in Table 1, under salinity stress, adecrease in Ca^2+^ content is observed in all tested variants. Nevertheless, the decline was more marked at 200 mM NaCl, reaching about 64.6% and 27.7% in roots, and 61.5% and 22.2% in shoots in *H. vulgare* and *H. marinum*, respectively, compared to control plants. Priming seeds with SA or CaCl_2_ provided an increase in the content of this nutrient in both species under the different salt concentrations compared to their unprimed ones. This positive effect was especially more noticeable in primed plants exposed to 100 mM NaCl (Table 1).

#### 2.1.3. Magnesium

Salinity in the culture media diminished Mg^2+^ content in plants of both barley species. However, the decrease was smaller in *H. marinum* plants than in *H. vulgare* where the decrease in Mg^2+^ reached 42% and 57.7%, respectively, in roots and shoots of plants grown with 200 mM NaCl, compared to control plants. Regarding *H. marinum* plants, under 200 mM NaCl treatment, the decline in Mg^2+^ contents wasabout 22% and 30%, respectively, in roots and shoots, in comparison with the control plants (Table 1). Interestingly, both priming agents were able to mitigate the salt-induced Mg^2+^ decreasein both species subjected to the salt treatments. In fact, when *H. vulgare* plants were exposed to 100 mM NaCl, CaCl_2_ provided the best improvement in shoot Mg^2+^ contents. However, both agents increased Mg^2+^ concentrations in roots, compared to unprimed stressed plants. When *H. marinum* plants were exposed to 200 mM NaCl as opposed to unprimed stressed plants, the stimulator effect of the priming agents was more noticeable. Indeed, the increase in Mg^2+^ concentration reached 28.5% and 47.7% in shoots from plants derived from primed seeds with SA and CaCl_2_, respectively. Additionally, plants grown from CaCl_2_ and SA-primed seeds showed increases inMg content in roots of 10% and 22.5%, respectively.

#### 2.1.4. Sodium

Along with the rise in salt concentration, salinity in the culture media caused a further increase in Na^+^ concentration in both species. Our data showed that the Na^+^ increase in roots and shoots was extremely pronounced in *H. vulgare* plants grown under 200 mM, compared to the control. It is important to mention that lower Na^+^ concentrations were registered in shoots of *H. marinum* plants subjected to either 100 or 200 mM NaCl (Table 2). Interestingly, CaCl_2_ and SA supplementation alleviated the salt-induced Na^+^ accumulation in plants from both species derived from primed seeds, compared to unprimed ones. SA priming was more efficient when *H. vulgare* plants were exposed to 200 mM NaCl and provided a decrease in Na content in shoots and roots by 35.3% and 31%, respectively, compared to unprimed plants grown with this same salt treatment. Seed priming with SA was also the best treatment to alleviate Na^+^ accumulation in *H. marinum* plants. In this case, SA reduced Na^+^ contents in shoots by 18% in the presence of 100 mM NaCl and by 32% in roots from plants grown in the presence of 200 mM NaCl, in relation to their unprimed NaCl-treated controls (Table 2).

#### 2.1.5. Potassium

As expected, salinity decreased K^+^ levels in both species progressively with the increasing salinity level (Table 2). The decrease in K^+^ concentration was more pronounced when plants of both species were exposed to 200 mM NaCl, reaching decreases of 51.7% and 27% in roots and 37% and 25.3% in shoots, respectively, in *H. vulgare* and *H. marinum* plants, compared to the control. However, it is important to note that concentrations of this nutrient are considerably higher in *H. marinum* than in *H. vulgare* plants under saline stress. Under restrictive conditions, seed priming improved K^+^ concentration in both species. For *H. vulgare*, SA was the most efficient treatment providing thebest K^+^ uptake, leading to 30% and 46% increases in shoots and roots, respectively, in the presence of 200 mM NaCl, as compared to the unprimed plants treated with 200 mM NaCl (Table 2). Regarding *H. marinum*, similarly, both agents enhanced K^+^ content in shoots and roots of plants derived from primed seeds, compared to unprimed plants when they were exposed to both salt treatments (Table 2).

#### 2.1.6. Sodium/Potassium Ratio

In the absence of priming, the Na^+^/K^+^ ratio, which provides information about the potential of the plants to discriminate the two ions, was stimulated by salinity in both barley species. However, this stimulation was clearly marked in *H. vulgare* plants. Particularly, in plants exposed to 200 mM NaCl, the percentage of increase reached its maximum in comparison with NaCl-untreated plants (Table 2). Regarding *H. marinum*, the Na^+^/K^+^ ratio was also stimulated by salt stress but to a lesser extent than in *H. vulgare* plants (Table 2). Interestingly, seed priming with both agents decreased this ratio in both species grown under both salt concentrations. Among them, SA had the best beneficial effect (Table 2).

### 2.2. Effect of SA and CaCl_2_ Seed Priming on Membrane Integrity under Salinity

#### 2.2.1. Electrolyte Leakage

**Figure 1:**
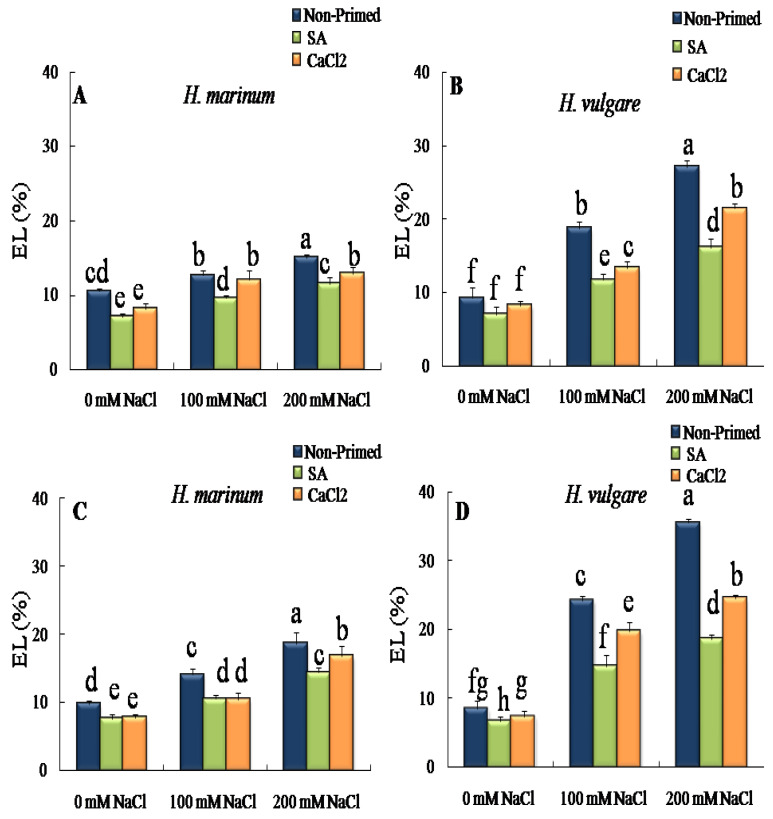
Effect of SA and CaCl_2_ seed priming on the electrolyte leakage (EL) levels in leaves (**A**,**B**) and roots (**C**,**D**) in two barley species, *H. vulgare* and *H. marinum*, subjected to control (0 mM NaCl) and two salt concentrations regimes (100 and 200 mM NaCl). Values are means of 5 independent replicates ± standard error. Different letters indicate significant differences according to the Tukey test at *p* < 0.05.

The findings demonstrated that electrolyte leakage was considerably elevated by salinity in plants of both species of barley grown from untreated seeds (Figure 1). This increase depended on the species and the applied salt concentration and it was rather noticeable in *H. vulgare*. In this species, NaClstress progressively increased EL, especially in leaves. In the presence of 200 mM NaCl, EL increases around 4 fold in leaves and around 3 fold in roots (Figure 1). In contrast, the effect of 200 mM NaCl in EL in *H. marinum* was less marked. In this case, EL rises by 43% in leaves and by 89% in roots, compared to their respective controls grown from non-pre-treated seeds subjected to 200 mM NaCl (Figure 1B,D). However, in *H. vulgare*, pre-treatment with SA or CaCl_2_ significantly decreased EL levels in plants grown in the presence of NaCl stress. This decrease was more noticeable in plants derived from SA-pre-treated seeds and exposed to 200 mM NaCl. Under these conditions, SA pre-treatment decreased EL values by 21% and 31% in leaves and roots, respectively, compared to their counterparts grown from non-pre-treated seeds (Figure 1A,C). In *H. marinum*, both agents slightly decreased EL values in the leaves and roots of plants subjected to different salt concentrations (Figure 1B,D).

#### 2.2.2. Lipid Peroxidation

**Figure 2:**
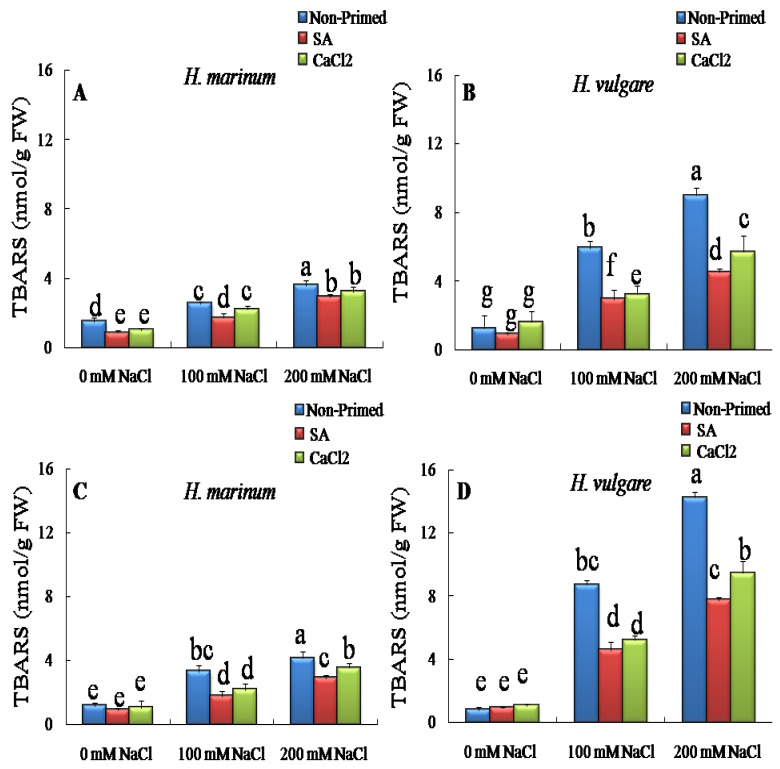
Effects of SA and CaCl_2_ seed priming on the thiobarbituric reactive substances content (TBARS) in leaves (**A**,**B**) and roots (**C**,**D**) in two barley species, *H. vulgare* and *H. marinum*, subjected to control (0 mM NaCl) and two salt concentrations regimes (100 and 200 mM NaCl). Values are means of 5 independent replicates ± standard error. Different letters indicate significant differences according to the Tukey test at *p* < 0.05.

Figure 2 shows that, in the absence of pre-treatment, salinity induced a significant increase in TBARS levels in both barley species in a concentration-dependentmanner. Indeed, this increase was particularly noted in *H. vulgare* plants grown in the presence of 200 mM NaCl (Figure 2A,C). Indeed, the increase of TBARS levels reached 13 fold in leaves and 5 fold in roots in the presence of 200 Mm NaCl (Figure 2A,C). In *H. marinum*, the increase in TBARS content was much smaller than that observed in *H. vulgare*, reaching 140% and 250%, in leaves and roots, respectively, of plants subjected to 200 mM NaCl, compared to plants grown from non-pre-treated seeds subjected to 200 mM NaCl (Figure 2B,D). Seed priming with both SA and CaCl_2_ significantly decreased the TBARS content in leaves and roots from *H. vulgare* plants under both salinity treatments. This decrease was more noticeable in plants subjected to 200 mM NaCl from SA-pre-treated seeds, compared to those from non-pre-treated seeds (Figure 2A,C). It is important to note that in the leaves and roots ofplants exposed to 100 mM NaCl, the two agents have a similar effect. In *H. marinum*, the effect of SA and CaCl_2_ treatments on the TBARS content was less significant in stressed plants compared to the effects observed in *H. vulgare*. Furthermore, the effect of SA and CaCl_2_ on TBARS levels was similar, regardless of the applied salt concentration (Figure 2B,D).

### 2.3. Effect of SA and CaCl_2_ Seed Priming on Enzymatic Antioxydant Activities under Salinity

#### 2.3.1. Catalase

In the absence of NaCl, *H. vulgare* plants showed lower CAT activity than *H. marinum* plants, both in leaves and roots (Table 3). In the presence of NaCl treatments, CAT increased in a concentration-dependent manner in *H. marinum*, but not in *H. vulgare* (Table 3). The increase is about 4 times in both leaves and roots of plants subjected to 200 mM NaCl compared to the control. However, pre-treatment with SA and CaCl_2_ significantly induced catalase activity only in *H. vulgare* plants. This induction was more pronounced in plants derived from SA-pre-treated seeds subjected to 200 mM NaCl, withanincrease of 9 fold in leaves and 15 fold in roots, compared to plants grown from unprimed seeds.

#### 2.3.2. Superoxide Dismutase

Compared to *H. marinum*, *H. vulgare* has lower constitutive SOD levels under control conditions. On the other hand, plants of both species exhibited significantly increased SOD activity in response to salinity stress (Table 3). Specifically, *H. marinum* plants grown on a culture medium supplemented with 200 mM showed a higher increase in SOD activity (Table 3). This increase reached 350% in leaves and 360% in roots of plants subjected to 200 mM NaCl compared to the control. Interestingly, SA and CaCl_2_ treatment produced a different effect depending on the plant species, while priming treatment induced a significant increase in SOD activity in *H. vulgare*, it decreased its ativity in *H. marinum* (Table 3). Indeed, the highest SOD activity was noted in *H. vulgare* plants grown from seeds pre-treated with CaCl_2_ and subjected to 200 mM, reaching increases of 460% and 360% in leaves and roots, respectively, compared to their counterparts grown from non-pre-treated seeds (Table 3).

#### 2.3.3. Peroxidase

Under control conditions, *H. vulgare* registered lower constitutive POX levels than *H. marinum*. Nevertheless, salinity stimulated significantly POX activity in plants of both species (Table 3). In particular, the highest increase in POX activity was extremely noted in *H. marinum* plants grown on a culture medium supplemented with 200 Mm NaCl (Table 3). It is interesting to note that under the influence of both salt treatments, POX activity was considerably elevated in plants of both barley species when SA and CaCl_2_ seed priming was implicated. In contrast to plants grown from non-pre-treated seeds, the greatest POX increase was observed in *H. vulgare* plants grown from SA-pre-treated seeds and subjected to 200 mM NaCl (Table 3).

### 2.4. Pearson’s Correlation Matrix Analysis

Results shown for the correlations matrix are concomitant with those obtained from the trait-by-trait analyses. In fact, we noticed a significant effect of all parameters measured in plants grown from unprimed seeds of *H. vulgare* species subjected to 200 mM NaCl treatments (Table 4). In the current work, the most marked result of this salt treatment is the reduction of Fe (r = 0.704 and 0.637), Ca^2+^ (r = −0.752 and −0.68), Mg^2+^ (r = −0.76 and −0.58) and K^+^ (r = −0.68 and −0.662) contents, respectively, in shoots and roots. Moreover, Na^+^ content (r = 0.887 and 0.68) and Na^+^/K^+^ ratio (r = 0.89 and 0.808) were found to be reduced in plants grown under this same salinity concentration. In concomitance with these results, 200 mM NaClsalt treatment initiated a great increase in EL values (r = 0.779 and −0.842) and TBARS contents (r = 0.818 and 0.829) in shoots as well as roots, respectively (Table 4), which was less pronounced for *H. marinum* plants (r = 0.643 and 0.652 for EL) and (r = 0.621 and 0.614 for TBARS), respectively, in shoots and roots (Table 5). Furthermore, data fromthe correlations matrix confirmed the prominent increase in enzymatic activity in *H. marinum* plants grown from unprimed seeds, particularly subjected to 200 mM NaCl. Indeed, CAT activity (r = 0.595 and 0.835), SOD activity (r = 0.884 and0.855) and POX activity (r = 0.795 and 0.784) were stimulated under salinity conditions (Table 5).

## 3. Discussion

Osmotic stress, mineral nutrient imbalance, ionic toxicity and physiological and biochemical disruptions are some of the effects of salinity on plants. Data fromthe current study revealed that salinity decreased Fe content in the shoots and roots of both barley species, which is consistent with results found in other plants [31]. The fact that saline treatments inhibited Fe transport in shoots by competing with Na^+^ may help to explain this effect. It has been demonstrated in this context that salinity alters the mineral relations of plants by competing with Na^+^ to reduce the availability of mineral nutrients [7]. This agrees with our results in which, under NaCl stress, *H. vulgare* registered the lowest K, Mg, Ca and Fe concentrations in roots and shoots. Interestingly, priming seeds with SA and CaCl_2_ strongly improved the maintenance of optimal mineral nutrition. Our results supported previous reports, which explained the ameliorative effect of SA pre-treatment by the powerful capacity of this phytohormone to stimulatemitotic activity [32]. Moreover, SA stimulatesnutrient uptake and transport into plants via the induction of plasma membrane H^+^-ATPase activity, which plays an important role in the transport of essential elements [33]. A similar trend was observed for K^+^, Mg^2+^ and Ca^2+^ in wheat plants exposed to salt stress [34]. In this same context, Khan et al. (2010) exhibited that the treatment of mung bean plants with 0.5 mM 0.5 SA results in a maximum decrease in Na content, while K^+^, Mg^2+^ and Ca^2+^ contents increased under saline conditions [22]. From this angle, it has been noted that SA improved the uptake of Fe in soybean plants cultivated in calcareous soils [35].

In addition, it is worth mentioning that excessive Na^+^ ions present in the nutrition solution induced its accumulation, especially in shoots of *H. vulgare*. Excessive accumulation of Na^+^ and Cl^−^ in plants grown under saline conditions leads to ionic imbalance, specific ionic effects and symptoms of nutritional deficiencies in plants [36]. The results showed that Na accumulation was increased by salt treatment, while it was decreased by combined SA or CaCl_2_ seed priming and salt treatments. Similar results were obtained for *Ocimumbasilicucm* [37] and broad bean [38]. However, K^+^ content was significantly reduced by salt treatments and increased when salt and seed priming treatments with both agents were combined. As predicted by Gong et al. (2013) for tomatoes and Li et al. (2014) for wheat, plants grown under saline stress showed a notable decrease in K^+^ concentration. It has also been proposed that there may be less competition for the uptake of K^+^ and Na^+^ due to a decrease in the uptake system’s performance [39,40]. Jayakannan et al. (2013) showed that the application of SA under salt stress conditions increased K^+^ content and decreased Na^+^ accumulation in Arabidopsis shoots [41]. This may be attributed to the effects of Na^+^ and K^+^ transport in plant cells due to their chemical similarity [37]. Considered together, the positive impact of SA and CaCl_2_ on barley growth could be ascribed to the preservation of the greatest mineral uptake of mineral elements [42]. The effect of SA on plant mineral nutrition under salt stress can be different depending on the mode of application. The SA treatment of NaCl-treated pea plants by spraying produced no important changes in leaf nutrient content; however, the treatment with 100 µM SA produced a decrease in the Na contents in roots [43].

Plants of both barley species grown from the pre-treated and non-pre-treated seeds showed significant variability in response to salinity constraint. This variability was expressed both in terms of levels of EL and TBARS content as an oxidative stress parameters, and antioxidant enzyme activities. It is generally known that salinity induces the production of ROS in plants at the subcellular level [9,10]. These ROS lead to changes in the structural and chemical properties of cell membranes [44]. In this experiment, we found that salinity causes a significant increase in TBARS levels in plants of both barley species. This deleterious effect was more pronounced in plants of the less NaCl-tolerant species *H. vulgare* subjected to the higher salt concentration. In this sense, the stimulation of TBARS content was defined as a good indicator of oxidative stress [45], which is a parameter that indicates membrane damage [46]. Indeed, the results obtained are comparable to those found in other plants subjected to salinity [47,48,49]. In addition, increased lipid peroxidation has been associated with salinity damage in tomatoes [39]. Similar results were found by Sánchez-Rodríguez et al. (2010), showing that EL and TBARS increased under stress conditions [50]. The results obtained in this study confirm those observed in various plant species [47,51].

The constitutive levels for CAT, SOD and POX activities were higher in *H. marinum* than in *H. vulgare*, both in leaves and roots. Different researchers have shownthat the response of antioxidant defenses in salt-tolerant species is different from salt-sensitive species. Some authors have observed that the species more tolerant to salinity had a higher constitutive level of antioxidant enzymes [52,53,54]. In contrast, in salt-sensitive species, antioxidant defenses show an unchanged response or even decrease [47]. In the present work, we also observed that the effect of salt stress on antioxidant enzymes depends on the barley species analyzed. In that regard, in *H. marinum*, more NaCl-tolerant than *H. vulgare*, the activity levels of CAT, SOD and POX showed an important and progressive increase by the effect of NaCl stress in both organs. However, in *H. vulgare*, the observed NaCl-induced increases in SOD and CAT were less important in comparison to *H. marinum* plants. Our findings concur with those of Hernandez et al. (2001), who demonstrated that plants have multiple defense mechanisms against ROS, including a number of antioxidant enzymes such as superoxide dismutase, catalase, peroxidase and the ascorbate-glutathione cycle enzymes that enable the preservation of a high antioxidant capacity [55]. Additionally, Esfandiari et al. (2007) discovered that salt stress raised wheat’s SOD activity, one of several significant antioxidant enzymes capable of repairing oxidative damage brought on by ROS. Therefore, SOD is thought to be a key enzyme in managing oxidative stress in maintaining normal physiological conditions and regulating intracellular superoxide levels. In that regard, a correlation between SOD activity and decreased oxidative damage has been described [56,57]. The increased CAT activity was reported in tomatoes and mulberriesin response to salt stress [58,59]. Similarly, Tahjib-Ul-Arif et al. (2018) concluded that increased catalase activity enabled ROS scavenging in maize plants grown under salinity stress [60].

The effect of SA and CaCl_2_ treatments on the activity of the antioxidant enzymes is also different in both plant species. Thus, in *H. marinum*, both treatments reduced the activity levels of the analyzed antioxidant enzymes when plants were growingin the presence of NaCl, whereas the opposite was observed in *H. vulgare*. However, the SA and CaCl_2_-induced declinesof EL and TBARS contents, as well as the high enzymatic antioxidant activities, indicate that both agents could significantly mitigate the adverse effects of salinity and alleviate the oxidative stress generated by this environmental stress in barley plants. There was a reduction in TBARS levels and EL levels in plants derived from pre-treated seeds compared to those derived from non-pre-treated seeds. This beneficial effect was significantly noted in *H. vulgare* species. Thus, pre-treatments mitigated the effects of salinity by modulating the activity of various antioxidant enzymes. In the same perspective, several researchers proved the crucial role of SA and CaCl_2_ in the protection of cellular membranes, reflected in decreasing TBARS and EL levels. As SA decreased levels of lipid peroxidation in different plant species, it has been suggested that it may act as an ROS scavenger [61]. Furthermore, it was proved in a previous study that the application of SA induced an antioxidant defense response by stimulating many of the antioxidant enzymes required to protect crops from osmotic, salinity and other stresses [62,63]. Our results are comparable to those found by other authors who showed that SA treatment increased catalase, POX and SOD activities [64,65,66]. Also, El-Esawiet al. (2017) proved that SA enhanced the gene expression of SOD in *Rosmarinus officinalis* plants subjected to salinity [67]. Again, it has been suggested by Ahmed et al. (2020) that foliar application of SA also resulted in a significant increase in nutrient use efficiency that correlated with significant changes in POX and SOD activities [68]. The increase in antioxidant enzyme activities by SA suggests that plants can be more effective in ROS detoxification. However, the pre-treatment of seeds with SA in *H. marinum* species induced a decrease in enzyme activities in plants subjected to salt stress, although plants maintained good SOD activity levels in the presence of 200 mM NaCl, even higher thanthe values observed In *H. vulgare* under the same conditions. Our results are in agreement with those of Barba-Espín et al. (2010), who noted that exogenous application of SA does not always have a positive effect on the improvement of oxidative stress under restrictive conditions. Its stimulating effect can be dependent on the applied stress, the mode and the time of SA application and the species studied [69]. Similarly, Joshi et al. (2013), studying the effect of CaCl_2_ pre-treatment of cucumber (*C. sativus* L.) seeds on germination and plant growth in a salt-supplemented medium, proposed that the observed positive impact may be associated with the activation of antioxidant systems [28]. Similar measurements were recorded in pepper. Thus, seed priming with CaCl_2_ seems to play a crucial role in the establishment of basal resistance to environmental constraints.

*H. marinum* contained higher constitutive levels of the antioxidant enzymes both in leaves and roots. In addition, SOD, CAT and POX activity were stimulated by NaCl stress, and thus were able to better cope with the possible increase in ROS accumulation under saline conditions, which is reflected in less damage to the membranes. This can partially explain thebetter plant performance of *H. marinum* than *H. vulgare* under saline conditions. However, the priming treatments increased the activity levels of the antioxidant enzymes in leaves and roots from *H. vulgare* plants, which would allow these plants to cope with the salt-induced oxidative stress. Probably, *H. vulgare* plants could induce the photorespiratory pathway, as observed by the CAT stimulation in leaves, which can have a role in dissipatingthe excess reducing power accumulation in the photosynthetic electron transport chain, thus protecting the chloroplast under saline stress challenge, as described by other authors in salt-stressed plants [70,71].

## 4. Materials and Methods

### 4.1. Plant Growth Conditions and Treatments

Seeds of both barley species: *H. vulgare* L. ‘Manel’ (cultivated species) were provided by the National Institute of Agronomy of Tunis (INAT) and *H. marinum* Huds. (Wild species) were collected from Sebkha Kalbia (an intermittent in Tunisia that covers 8000 hectares in Sousse governorate at 35°5003400′ north, 10°1601800′southeast of Kondar). Uniform barley seeds were selected and disinfected for 8 min with sodium hypochlorite (2%, *v*/*v*) and then soaked separately for 20 h either in distilled water (unprimed seeds), SA (1.25 mM) or CaCl_2_ (5 mM) solution. Thereafter, unprimed and primed seeds were germinated for 7 days at 20 °C in Petri dishes. After that, seven-day-old seedlings were moved to a half-strength aerated nutrient solution for 7 days. Seedlings were divided into 3 groups. The first group corresponded to control plants (derived from unprimed seeds), the second one to plants derived from seeds primed with SA andthe third one to plants derived from seeds primed with CaCl_2_. Subsequently, similar-sized seedlings were selected and distributed in groups as mentioned above grown in full-strength aerated medium solutions for 15 days. At this time, when plants were at the third leaf stage, 3 salt treatments were applied (0, 100 and 200 mM NaCl) for each of the 3 groups of plants for15 days. Each group contained 72 plants (8 plants per container of 5 L) (9 replicates per treatment). The nutritive solution for all treatments is prepared as follows: The macronutrient (mM) composition was 1.5 MgSO_4_, 1.6 KH_2_PO_4_, 0.6 K_2_HPO_4_, 3 KNO_3_, 3.5 Ca (NO_3_)_2_ and 2 NH_4_NO_3_. However the micronutrients (mg L^−1^) were MnSO_4_ (0.5), CuSO_4_ (0.04), ZnSO_4_ (0.05), H_3_BO_3_ (0.5) and Mo_7_O_24_(0.02). Fe was supplied as Fe (III)–EDTA formula. All aerated hydroponic cultures were carried out under controlled conditions in a glasshouse under a luminous ceiling with an effective radiation of 250 µmolm^−2^ s^−1^ and a photoperiod of 16 h/8 h (light/dark). The average temperature and relative humidity were 24 °C/60% during the day and 18 °C/80% at night.

### 4.2. Nutrient Extraction and Analysis

Forty mg of dried vegetal material was ground and digested with 15 mL in sulfuric acid (H_2_SO_4_, 1N) for 1 h at 80 °C and then left overnight at room temperature. This allows the total extraction of the target elements of samples. The different cations (Fe^2+^, Mg^2+^ and Ca^2+^) were analyzed using an atomic absorption spectrophotometer (Perkin Elmer, Analyst 300, Rodgau, Germany). However, K^+^ and Na^+^ ions are determined by flame spectrophotometer (BWB Technologies XP, ES, Garhes, Fr). Results are expressed in μg·g^−1^DW for Fe^2+^ and in mg·g^−1^DW for Mg^2+^, Ca^2+^, K^+^ and Na^+^ [72].

### 4.3. Electrolyte Leakage

Using a digital conductivity meter (BANTE instruments 950, Shanghai, China), the membrane stability index (MSI) was measured in order to estimate electrolyte leakage. For every treatment, ten replicates were carried out. After cutting 0.2 g of fresh material, it was heated for 30 min at 32 °C in a falcon tube with 10 mL of distilled water, and the electrical conductivity (EC1) was then measured. Following two hours of keeping the sample at 100 °C, the electrical conductivity (EC2) was also measured. In accordance with Dionioese and Tobita, the MSI was computed using Equation [73]:

(1) $$\mathrm{MSI}=\left(\frac{\mathrm{EC}1}{\mathrm{EC}2}\right)*100$$

where EC1—electrical conductivity measured at the first time and EC2—electrical conductivity measured at the second time.

### 4.4. Lipid Peroxidation

According to Bueg and Aust [74], lipid peroxidation was quantified as the 2-thiobarbituric acid-reactive substances (TBARS), primarily malondialdehyde. Using a pre-chilled mortar and pestle, frozen samples (1 g of fresh material, five replicates for each treatment) were homogenized with 10 mL (0.1%; *p*/*v*) of trichloroacetic (TCA) acid. The samples were centrifuged for ten minutes at 25°C to 10,000× *g*. Then, 4 mL of thiobarbituric acid (TBA) (0.5%; *p*/*v*) in 20% TCA was mixed with 1 mL of the upper liquid layer (supernatant). The absorbance of the supernatant (532 nm) was measured after centrifugation at 1000× *g* for 10 min, and the value corresponding to non-specific absorbance (600 nm) was subtracted. Equation was used to determine the TBARS concentration based on the molar extinction coefficient (155 mM^−1^ cm^−1^):

(2) $$\mathrm{TBARS}\left(\frac{\mathrm{nm}}{g\;\mathrm{FW}}\right)=\frac{\left(\mathrm{DO}532-\mathrm{DO}600\right)*\mathrm{VS}}{0.155\;}*\mathrm{FW}.$$

### 4.5. Antioxidant Enzyme Determinations

Catalase was evaluated using the spectrophotometric method described by Aebi (1984), which is based on the magnitude of the decrease in absorption at 240 nm caused by the disappearance of H_2_O_2_ [75]. SOD was assayed by the ferricytochrome c method using xanthine/xanthine oxidase as the source of superoxide radicals (McCord and Fridovich, 1969) [76]. Class III peroxidase activity was analyzed using 50 mm Tris-acetate buffer (pH 5.0), 0.5 mm H_2_O_2_ and 4-methoxy-α-naphthol (ε595 21.600 M^−1^cm^−1^) as electron donor (Ros-Barceló et al., 1998) [77].

### 4.6. Statistical Analysis

Statistical analyses were performed with the “XLSTAT” software (version 2014). The mean values and the standard error (SE) were obtained from at least 5 replicates for all physiological parameters analyzed and were analyzed using Tukey’s multiple range tests. A *p*-value less than 0.05 was considered statistically significant. Pearson’s correlation analysis was carried out based on principal component analysis (PCA) using XLSTAT software, considering the variables centered on their means and normalized with a standard deviation of 1.

## 5. Conclusions

In general, salinity affects mineral nutrition and antioxidant capacity differently in both barley species. Nevertheless, in *H. marinum*, the higher salt tolerance behavior was correlated with higher constitutive levels of antioxidant enzymes, which can contribute to maintaining reduced TBARS and EL values, as compared to the cultivated species *H. vulgare*. Seed priming, with SA and CaCl_2_, alleviated the deleterious effects of this abiotic stress by significantly increasing Fe, Ca^2+^, Mg^2+^ and K^+^ contents, decreasing Na^+^ content and Na^+^/K^+^ ratio, correlating with reduced TBARS and EL and antioxidant enzyme activities stimulation, suggesting an improved ROS detoxification capability under saline conditions. Thus, seed priming with SA or CaCl_2_may therefore confer an improvement in the salinity tolerance in *H. vulgare* species and ameliorate its growth under salinity conditions.

## Figures and Tables

**Table 1:** Effect of SA and CaCl_2_ seed priming on total Fe, Ca^2+^ and Mg^2+^ concentration in roots and shoots of plants in two barley species: *H. vulgare* and *H. marinum* exposed to control (0 mM NaCl) and two salt treatments (100 and 200 mM NaCl). Values are means of 10 independent replicates ± standard error. Different letters indicate significant differences according to the Tukey test at *p* < 0.05.

	Treatments	Control	100 mM NaCl	200 mM NaCl	100 mM NaCl+ SA	200 mM NaCl+ SA	100 mM NaCl+ CaCl_2_	200 mM NaCl+ CaCl_2_
Parameters								
*H. vulgare*								
Shoot Fe (mg/gDW)		1468 ^a^	898 ^d^	608 ^e^	1264 ^b^	916 ^d^	1117 ^c^	782 ^d^
Root Fe (mg/g DW)		1174 ^a^	777 ^c^	515 ^e^	1063 ^b^	780 ^c^	1049 ^b^	651 ^d^
Shoot Ca^2+^ (mg/g DW)		0.421 ^a^	0.286 ^c^	0.162 ^e^	0.354 ^b^	0.294 ^c^	0.343 ^b^	0.230 ^d^
Root Ca^2+^ (mg/g DW)		0.339 ^a^	0.220 ^d^	0.120 ^f^	0.311 ^b^	0.191 ^e^	0.272 ^c^	0.185 ^e^
ShootMg^2+^ (mg/gDW)		0.238 ^a^	0.163 ^d^	0.101 ^e^	0.205 ^c^	0.162 ^d^	0.223 ^b^	0.160 ^d^
Root Mg^2+^ (mg/gDW)		0.263 ^a^	0.205 ^c^	0.152 ^e^	0.244 ^a^	0.195 ^d^	0.271 ^a^	0.229 ^b^
*H. marinum*								
Shoot Fe (mg/gDW)		2206 ^a^	2141 ^b^	1758 ^e^	2159 ^b^	2084 ^c^	1862 ^d^	1848 ^d^
Root Fe (mg/gDW)		2421 ^a^	2263 ^b^	1919 ^e^	2310 ^b^	2199 ^c^	2195 ^c^	2012 ^d^
Shoot Ca^2+^ (mg/gDW)		0.863 ^a^	0.804 ^b^	0.671 ^c^	0.854 ^a^	0.793 ^bc^	0.842 ^ab^	0.684 ^c^
Root Ca^2+^ (mg/gDW)		0.883 ^a^	0.724 ^d^	0.638 ^f^	0.810 ^b^	0.690 ^e^	0.771 ^c^	0.684 ^e^
ShootMg^2+^ (mg/gDW)		0.372 ^a^	0.353 ^ab^	0.260 ^c^	0.370 ^ab^	0.344 ^ab^	0.334 ^b^	0.384 ^a^
RootMg^2+^ (mg/gDW)		0.354 ^a^	0.323 ^b^	0.275 ^d^	0.355 ^a^	0.337 ^b^	0.331 ^b^	0.303 ^c^

**Table 2:** Effect of SA and CaCl_2_ seed priming on Na^+^, K concentration and Na^+^/K ratio in roots and shoots of plants in two barley species: *H. vulgare* and *H. marinum* exposed to control (0 Mm NaCl) and two salt treatments (100 and 200 mM NaCl). Values are means of 10 independent replicates ± standard error. Different letters indicate significant differences according to the Tukey test at *p* < 0.05.

	Treatments	Control	100 mM NaCl	200 mM NaCl	100 mM NaCl+ SA	200 mM NaCl+ SA	100 mM NaCl+ CaCl_2_	200 mM NaCl+ CaCl_2_
Parameters								
*H. vulgare*								
Shoot Na^+^(mg/gDW)		0.078 ^g^	0.911 ^d^	1.485 ^a^	0.683 ^f^	0.961 ^c^	0.843 ^e^	1.024 ^b^
Root Na^+^ (mg/g DW)		0.065 ^f^	0.751 ^c^	1.129 ^a^	0.420 ^e^	0.778 ^c^	0.486 ^d^	0.937 ^b^
Shoot K^+^ (mg/g DW)		0.876 ^a^	0.727 ^b^	0.549 ^e^	0.786 ^b^	0.714 ^c^	0.732 ^c^	0.616 ^d^
Root K^+^(mg/g DW)		0.837 ^a^	0.647 ^c^	0.404 ^f^	0.817 ^a^	0.591 ^d^	0.730 ^b^	0.498 ^e^
Shoot Na^+^/K^+^		0.093 ^f^	1.254 ^c^	2.733 ^a^	0.873 ^e^	1.349 ^c^	1.152 ^d^	1.668 ^b^
Root Na^+^/K^+^		0.078 ^g^	1.167 ^d^	2.8 ^a^	0.517 ^f^	1.316 ^c^	0.668 ^e^	1.884 ^b^
*H. marinum*								
ShootNa^+^(mg/gDW)		0.095 ^f^	0.421 ^c^	0.515 ^a^	0.344 ^e^	0.459 ^b^	0.397 ^d^	0.479 ^b^
Root Na^+^ (mg/g DW)		0.101 ^g^	0.835 ^c^	1.060 ^a^	0.655 ^f^	0.716 ^e^	0.756 ^d^	0.893 ^b^
Shoot K^+^ (mg/g DW)		1.531 ^a^	1.269 ^b^	1.143 ^e^	1.303 ^b^	1.220 ^c^	1.243 ^b^	1.173 ^d^
Root K^+^ (mg/g DW)		1.484 ^a^	1.215 ^e^	1.084 ^f^	1.431 ^b^	1.260 ^d^	1.354 ^c^	1.189 ^e^
Shoot Na^+^/K^+^		0.062 ^e^	0.332 ^c^	0.451 ^a^	0.264 ^d^	0.376 ^b^	0.319 ^c^	0.421 ^a^
Root Na^+^/K^+^		0.068 ^f^	0.687 ^c^	0.978 ^a^	0.458 ^e^	0.568 ^d^	0.558 ^d^	0.752 ^b^

**Table 3:** Effect of SA and CaCl_2_ seed priming on the activity of catalase (CAT) (µmol min^−1^ mg^−1^ protein), superoxide dismutase (SOD) (U/mg protein) and peroxidase (POX) (nmol min^−1^ mg^−1^ protein) in roots and leaves of plants in two barley species: *H. vulgare* and *H. marinum* exposed to control (0 mM NaCl) and two salt treatments (100 and 200 mM NaCl). Values are means of 10 independent replicates ± standard error. Different letters indicate significant differences according to the Tukey test at *p* < 0.05.

	Treatments	Control	100 mM NaCl	200 mM NaCl	100 mM NaCl + SA	200 mM NaCl + SA	100 mM NaCl + CaCl_2_	200 mM NaCl + CaCl_2_
Parameters								
		*H. vulgare*						
CAT Leaves		5.7 ^f^	6.2 ^f^	9.3 ^e^	75.7 ^b^	97.2 ^a^	20.4 ^d^	52.6 ^c^
CAT Roots		5.2 ^f^	5.3 ^f^	8.2 ^e^	86.4 ^b^	136.5 ^a^	18.9 ^d^	63.8 ^c^
SOD Leaves		43.2 ^g^	52.8 ^f^	68.8 ^e^	123.8 ^c^	248.9 ^b^	107.4 ^d^	383.8 ^a^
SOD Roots		47.3 ^e^	48.9 ^e^	78.1 ^d^	120.3 ^c^	290.7 ^b^	118.9 ^c^	356.7 ^a^
POX Leaves		899 ^f^	1131.2 ^e^	1300.9 ^d^	41,287 ^c^	67,268 ^a^	38,263 ^c^	49,287 ^b^
POX Roots		1005 ^d^	999 ^e^	1330 ^d^	49,721 ^b^	58,055 ^a^	42,720 ^c^	50,223 ^b^
		*H. marinum*						
CAT Leaves		14.3 ^e^	51.7 ^b^	64.5 ^a^	23.9 ^c^	37.1 ^b^	7.1 ^d^	48.6 ^b^
CAT Roots		13.6 ^d^	48.2 ^b^	62.2 ^a^	33.1 ^c^	36.6 ^c^	31.5 ^c^	42.4 ^b^
SOD Leaves		68.6 ^d^	369.9 ^b^	849.3 ^a^	72.9 ^d^	369.2 ^b^	79.9 ^d^	277.1 ^c^
SOD Roots		78.9 ^d^	344.5 ^c^	824.7 ^a^	72.1 ^d^	406.4 ^b^	89.8 ^d^	344.2 ^c^
POX Leaves		1105 ^f^	26,720 ^b^	40,028 ^a^	14,055 ^d^	17,793 ^c^	1416 ^f^	12,289 ^e^
POX Roots		1277 ^d^	25,985 ^b^	41,204 ^a^	12,299 ^c^	22,436 ^b^	1359 ^d^	12,326 ^c^

**Table 4:** *H. vulgare* Pearson’s correlation matrix. Sh Fe: shoot iron content; R Fe: root iron content; Sh Ca^2+^: shoot calcium content; R Ca^2+^: root calcium content; Sh Mg^2+^: shoot magnesium content; R Mg^2+^: root magnesium content; Sh K: shoot potassium content; R K: root potassium content; Sh Na^+^: shoot sodium content; R Na^+^: root sodium content; Sh Na^+^/K: shoot sodium/potassium ratio; R Na^+^/K: root sodium/potassium ratio. Sh EL: shoot electrolyte leakage; R EL: root electrolyte leakage; Sh MDA: shoot malondialdehyde content; R MDA: root malondialdehyde content; Sh CAT: shoot catalase activity; R CAT: root catalase activity; Sh SOD: shoot superoxide dismutase activity; R SOD: root superoxide dismutase activity; Sh POX: shoot peroxidase activity; R POX: root peroxidase activity.

	C+100 mM NaCl	C+200 mM NaCl	SA+100 mM NaCl	SA+200 mM NaCl	CaCl_2_+100 mM NaCl	CaCl_2_+200 mM NaCl
Sh Fe	−0.096	**−0.704**	**0.669**	−0.058	**0.361**	−0.171
R Fe	−0.064	**−0.637**	**0.564**	−0.056	**0.532**	**−0.339**
Sh Ca^2+^	0.048	**−0.752**	**0.495**	0.103	**0.420**	**−0.315**
R Ca^2+^	0.024	**−0.680**	**0.667**	−0.179	**0.392**	−0.224
Sh Mg^2+^	−0.066	**−0.760**	**0.401**	−0.075	**0.603**	−0.102
R Mg^2+^	−0.101	**−0.580**	**0.258**	−0.189	**0.499**	0.113
Sh K^+^	0.196	**−0.680**	**0.486**	0.131	0.219	**−0.352**
R K^+^	0.101	**−0.662**	**0.635**	−0.073	**0.364**	**−0.366**
Sh Na^+^	−0.130	**0.887**	**−0.534**	−0.041	−0.251	0.069
R Na^+^	0.001	**0.680**	**−0.593**	0.050	**−0.474**	**0.334**
Sh Na^+^/K^+^	−0.182	**0.890**	**−0.457**	−0.113	**−0.255**	0.118
R Na^+^/K^+^	−0.129	**0.808**	**−0.503**	−0.043	**−0.416**	**0.282**
Sh EL	0.023	**0.779**	**−0.546**	−0.204	−0.355	0.304
R EL	0.089	**0.842**	**−0.590**	−0.280	−0.159	0.098
Sh MDA	0.152	**0.818**	**−0.491**	−0.152	−0.432	0.105
R MDA	0.054	**0.829**	**−0.525**	−0.082	−0.435	0.159
Sh CAT	**−0.486**	−0.446	0.418	**0.697**	−0.301	0.117
R CAT	−0.449	−0.421	0.312	**0.782**	−0.321	0.098
Sh SOD	−0.321	−0.290	−0.180	0.070	0.384	0.337
R SOD	**−0.469**	−0.355	−0.192	**0.468**	−0.199	**0.747**
Sh POX	**−0.586**	**−0.583**	0.153	**0.634**	0.097	0.285
R POX	**−0.624**	**−0.618**	0.298	0.465	0.169	0.310

Variables were centered around their means and normalized with a standard deviation of 1. n = 10. Values in bold represent significant correlations at the 0.05 level.

**Table 5:** *H. marinum* Pearson’s correlation matrix. Sh Fe: shoot iron content; R Fe: root iron content; Sh Ca^2+^: shoot calcium content; R Ca^2+^: root calcium content; Sh Mg^2+^: shoot magnesium content; R Mg^2+^: root magnesium content; Sh K: shoot potassium content; R K: root potassium content; Sh Na^+^: shoot sodium content; R Na^+^: root sodium content; Sh Na^+^/K: shoot sodium/potassium ratio; R Na^+^/K: root sodium/potassium ratio; Sh EL: shoot electrolyte leakage; R EL: root electrolyte leakage; Sh MDA: shoot malondialdehyde content; R MDA: root malondialdehyde content; Sh CAT: shoot catalase activity; R CAT: root catalase activity; Sh SOD: shoot superoxide dismutase activity; R SOD: root superoxide dismutase activity; Sh POX: shoot peroxidase activity; R POX: root peroxidase activity.

	C+100 mMNaCl	C+200 mMNaCl	SA+100 mMNaCl	SA+200 mMNaCl	CaCl_2_+100 mMNaCl	CaCl_2_+200 mMNaCl
Sh Fe	**0.443**	**−0.580**	**0.490**	**0.290**	**−0.302**	**−0.341**
R Fe	**0.329**	**−0.668**	**0.464**	0.142	0.131	**−0.398**
Sh Ca^2+^	0.159	**−0.560**	**0.427**	0.100	**0.365**	**−0.492**
RCa^2+^	0.033	**−0.609**	**0.678**	−0.220	**0.386**	**−0.268**
Sh Mg^2+^	0.083	**−0.565**	0.205	0.020	−0.049	**0.305**
R Mg^2+^	0.040	**−0.690**	**0.516**	0.245	0.160	**−0.273**
Sh K^+^	0.204	**−0.382**	**0.358**	−0.022	0.083	−0.240
R K^+^	−0.152	**−0.647**	**0.661**	0.017	**0.373**	−0.253
Sh Na^+^	−0.105	**0.576**	**−0.669**	0.168	**−0.282**	**0.312**
R Na^+^	0.053	**0.788**	**−0.539**	**−0.337**	−0.208	0.243
Sh Na^+^/K^+^	−0.166	**0.525**	**−0.560**	0.090	−0.242	**0.353**
R Na^+^/K^+^	0.054	**0.813**	**−0.547**	**−0.258**	**−0.283**	0.221
Sh EL	0.109	**0.643**	**−0.711**	−0.342	0.156	0.145
R EL	−0.057	**0.652**	**−0.538**	0.029	**−0.479**	0.393
Sh MDA	−0.117	**0.621**	**−0.671**	0.141	−0.338	0.363
R MDA	0.186	**0.614**	**−0.643**	−0.043	−0.414	0.300
Sh CAT	0.317	**0.595**	−0.362	−0.035	**−0.747**	0.232
R CAT	0.249	**0.835**	−0.391	−0.250	−0.466	0.023
Sh SOD	0.058	**0.884**	−0.454	0.056	−0.442	−0.102
R SOD	−0.003	**0.855**	**−0.493**	0.108	−0.463	−0.004
Sh POX	0.291	**0.795**	−0.172	−0.050	**−0.626**	−0.238
R POX	0.224	**0.784**	−0.243	0.114	**−0.634**	−0.246

Variables were centered around their means and normalized with a standard deviation of 1. n = 10. Values in bold represent significant correlations at the 0.05 level.

## Data Availability

Data are contained within the article.
